# Efficacy of FiLaC in different types of anal fistula. A systematic review and meta-analysis

**DOI:** 10.1007/s10103-026-05012-1

**Published:** 2026-09-01

**Authors:** Xingli Xu, Jiong Wu, Jialin Qin, Lei Jin, Zhicheng Li, Peter C. Ambe

**Affiliations:** 1 Shanghai University of Traditional Chinese Medicine, Shanghai, China; 2 Chair of Surgery II, Witten/Herdecke University, Witten, Germany

**Keywords:** FiLaC, Laser fistula tract closure, Fistula in ano, Anal fistula, Simple fistula, Complex fistula, Healing rate, Fistula closure

## Abstract

Fistula Tract Laser Closure (FiLaC) represents a minimally invasive technique for the management of fistula in ano. However, no data exists on the success rates of FiLaC for the different fistula types. This systematic analysis and meta-analysis were performed following the PICOS and PRISMA guidelines. A systematic literature review was conducted for articles published in Web of Science, Embase, Cochrane Library, PubMed using search terms ‘Anal fistula OR fistula in ano OR anorectal fistula’ (Mesh term), ‘Laser’, ‘Park´s classification, ‘complex fistula (Mesh term), and ‘Clinical Trial’ (Mesh term), ‘healing rate’, and ‘closure rate’ from inception until February 2025. Pooled results for 16 studies indicated higher healing rates for simple fistula (60.8%) compared to complex fistula (54.6%). Amongst complex fistulae, transsphincteric fistulae have the best odds of healing following FiLaC. The success rate of FiLaC for extrasphincteric and suprasphincteric fistulae is about 50%. Amongst all fistula types, although not statistically significant simple fistulae have a higher odds of healing following FiLaC in comparison to complex fistulae. Amongst complex fistulae, transsphincteric fistulae have the best success rates following FiLaC. Extrasphincteric and suprasphincteric fistulae have about 50% success rate following FiLaC.

**Supplementary Information:**

The online version contains supplementary material available at https://doi.org/10.1007/s10103-026-05012-1.

## Introduction

Anal fistula is a rather common condition, and surgical management is the standard of care [1–3]. However, surgical repair of anal fistula can be challenging depending on the complexity of the fistula [4–7]. The Park´s classification represents a widely accept nomenclature for grading fistula in ano, enabling possible differentiation between simple and complex fistulae [8]. The complexity of a fistula in ano is largely related with the probability of healing and the risk of continence disturbance following surgery [9, 10]. From an anatomical point of view, the amount of sphincter muscular complex involved by the fistula largely defines its complexity. As an accepted role, fistulae involving more than 30% of the external sphincter muscle or more than 50% of the internal muscle are complex in nature [11, 12]. This is usually the case with high transsphincteric and high intersphincteric fistulae.

Simple fistulae are easy to manage via lying open with high healing rates and supposedly neglectable risk of postoperative continence disturbance. Complex fistulae on the other hand require specific sphincter preserving techniques to ensure postoperative continence preservation. Such techniques have been shown to have lower healing rates compared to lying open [9]. Currently, Ligation of the Intersphincteric Fistula Tract (LIFT) [13] and advancement flap [14] represent commonly favored open sphincter preserving techniques for anal fistula repair. On the other hand, the Video Assisted Anal Fistula Treatment (VAAFT) [15] and Fistula Tract Laser Closure (FiLaC) [16, 17] have been increasingly used to manage complex anal fistulae.

In the FiLaC technique laser energy from a radially emitting fiber is used to destroy and obliterate the epithelized fistula tract [18]. Current data suggest healing rates from 20% to 80% following FiLaC [16, 19, 20]. This variation in healing rates reported is per se a reflection of the heterogeneity in the way the FiLaC procedure is performed [19]. Another issue that may influence the healing rate following FiLaC is the complexity of the fistula. The aim of this systematic review and meta-analysis is to investigate the effect of fistula complexity per Park classification on healing rate following FiLaC.

## Methods

This study was conducted in accordance with the Cochrane Handbook of Systematic Reviews and Meta-analysis ver. 6.2 and reported according to the PRISMA (Preferred Reporting Items for Systematic Reviews and Meta-Analyses) guideline. A systematic literature review was conducted for articles published in Web of Science, Embase, Cochrane Library, PubMed were identified using search terms ‘Anal fistula OR fistula in ano OR anorectal fistula’ (Mesh term), ‘Laser’, ‘Park´s classification, ‘complex fistula (Mesh term), and ‘Clinical Trial’ (Mesh term), ‘healing rate’, and ‘closure rate’ from inception until February 2025. This systematic review was also registered with PROSPERO: CRD420250655230.

### Inclusion and exclusion criteria

We conducted a systematic review and meta-analysis of observational studies. The meta-analysis was done on studies reporting success rates for FiLaC for different fistula types.

The inclusion criteria using the PICOS statement are as follows:

- P—patients undergoing FiLaC for anal fistula, categorized using Park´s classification.
- I—a group of patients who received FiLaC.
- C: Comparison of efficacy between different types of anal fistula (intersphincteric, transsphincteric, suprasphincteric and extrasphincteric) treated with FiLaC.
- O: Efficacy (Healing or closure rate or success rate).
- S: randomized or non-randomized clinical trials, observational studies (cohort or case-control) and case series; full-text report (including preprint).

The exclusion criteria were as follows: (1) articles reported in languages other than English; (2) studies using other closure techniques other than FiLaC; (3) studies without fistula classification; and (4) unpublished studies or abstracts.

### Classification of anal fistula

**Fig. 1:**
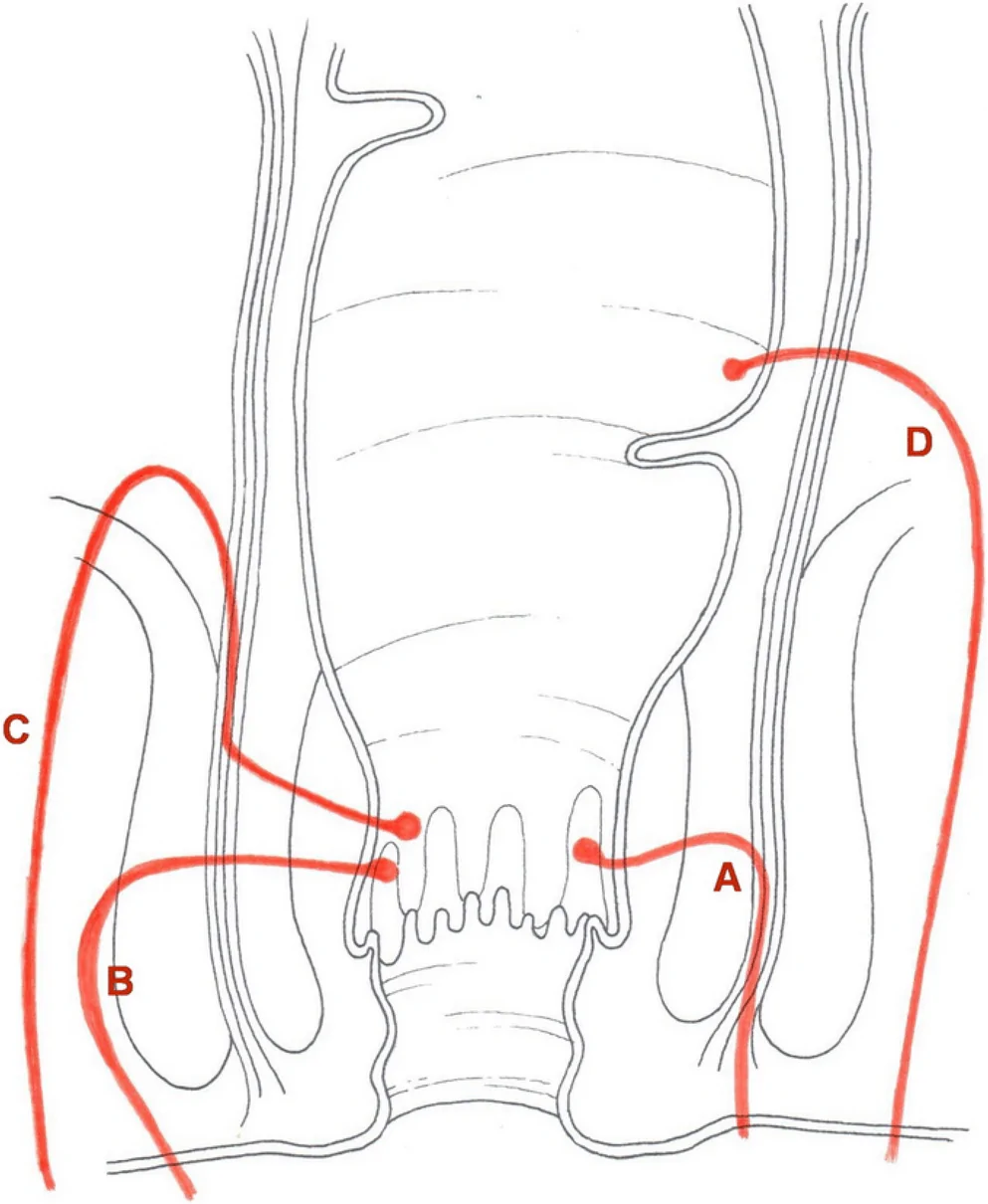
Different types of fistulae according to parks classification. **A**: Intersphincteric fistula **B**: transsphincteric fistula **C**: suprasphincteric fistula **D**: extrasphincteric fistula [21]

Park´s Classification, Fig. 1, was used in this study to characterize fistula complexity [8].

### Study selection and data extraction

Two independent investigators screened included articles according to the PRISMA guidelines for systematic reviews. The initial step was to screen the titles and abstracts to determine which articles met inclusion criteria. Hereafter, the references of relevant papers were scanned for possible eligible studies. Finally, full texts of included studies were independently analyzed by two investigators, and the inconsistencies are resolved through discussion and where needed in consensus with a third investigator.

### Statistical analysis

The Revman5.3 software provided by Cochrane Collaboration was used for data analysis. For dichotomous data, the risk ratio (RR) of 95% confidence interval (CI) is calculated, while for continuous data, average difference (MD) and standard average difference (95% CI) are calculated. Chi-square test and I^2^ statistics were used to evaluate the heterogeneity to determine whether the results of different studies were uniform.

### Assessment of risk of bias

The quality of the 15 non-RCTs’ quality was assessed using the Newcastle–Ottawa Scale [22, 23], while the remaining cross-sectional study was evaluated using the AHRQ (Agency for Healthcare Research and Quality) scale [24]. The details and results are shown in Table 1.

**Table 1:** Characteristics of the included studies

Author	Year	Country	Study Design	Age, years median(range)/ mean ± SD	Follow-up Time, months median(range)/ mean ± SD	Sex		Types of fistula*	Total Score
						Male	Female		
P. Giamundo [25]	2021	Italy	cohort studies	49(18–81)	6(9-120)	115	60	②③⑦	8
Lara Blanco Terés [26]	2023	Spain	cohort studies	48 ± 13.9	12(7–29)	20	16	① ②③⑥	8
A. Alam [27]	2019	France	cohort studies	32 ± 9.61	7.1(2-22.5)	10	10	②③④⑤⑥⑦⑧	7
Ömer Lütfi Akgül [28]	2021	Turkey	cohort studies	42(17–64)	24(6–30)	48	19	①②③⑥⑦⑧	8
Samuel Lalhruaizela [29]	2021	India	cohort studies	38.6 ± 11.5(18–64)	24 ± 24(12–36)	19	12	①②③④⑥⑦	8
Kursat R. Serin [30]	2020	Turkey	cohort studies	43.9 ± 12.9	11(6.0-17.6)	24	11	①②③⑤⑥⑧	8
G.de Bonnechose [31]	2020	France	cohort studies	43(22–88)	13.6(6–23)	60	32	② ⑥⑦	7
I. Marref [32]	2019	France	cohort studies	40(33–53)	6.3(4.2–9.3)	33	35	②③④⑤⑥⑦	8
A. Wilhelm [33]	2017	Germany	cohort studies	46(17–82)	25.4(6–60)	82	35	②③⑥⑦⑧	8
Ozgen Isik [34]	2020	Turkey	cohort studies	42(21–83)	48(6–56)	72	28	①②③④⑤⑥⑦	8
Ali Kemal Taskin [35]	2024	Turkiye	cross-sectional study	39 ± 11	17 ± 4(12–24)	24	6	① ②	8
J. Stijns [36]	2019	Netherlands	cohort studies	45(27–78)	10(IQR:7.3)	4	16	② ③	8
N. De Hous [37]	2019	Belgian	cohort studies	50(30–63)	9(4–26)	NA	NA(Total:103)	③ ③	6
Mustafa Cem Terzi [38]	2018	Turkey	cohort studies	43(18–78)	28.26(2.33–49.93)	82	21	① ②③⑥	8
Ersin Özturk [39]	2014	Turkey	cohort studies	41(23–83)	12(2–18)	37	13	①②③④⑤	8
Ismail Alper TARIM [40]	2021	Turkey	cohort studies	45 ± 14.5	13 ± 3.4	12	7	①②③	7

## Results

A total of 548 articles were retrieved from PubMed (146), Cochrane (24 trials), WOS (215), and Embase (163). After removing 203 duplicates, 345 articles remained. Following title and abstract screening, 125 articles were preliminarily included. After full-text review, 16 studies met the inclusion and exclusion criteria. The excluded articles were as follows: Abstract only, full text unavailable: 57, not related to FiLaC: 8, animal studies: 3, no classification of anal fistula or lacking relevant efficacy indicators: 21, review articles:12, editorials or commentaries: 4, and descriptive studies on FiLaC procedure only: 4, Fig. 2.

**Fig. 2:**
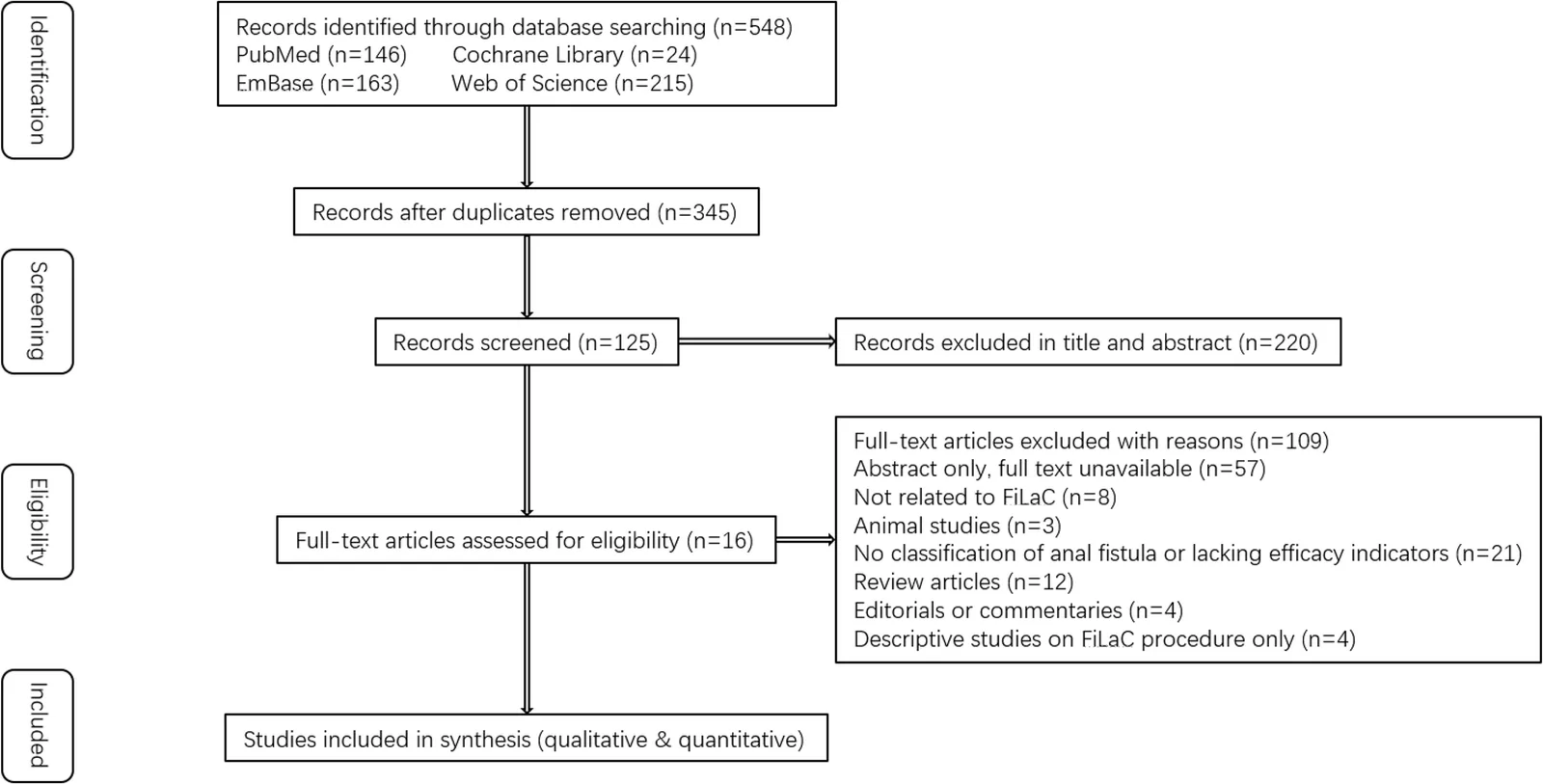
PRISMA Flowchart of included studies

Due to the lack of detailed reporting on complications, recurrence rates, and other relevant data for different types of anal fistula surgeries in the original studies, this analysis focused exclusively on primary healing data. The specific results of the meta-analysis are elaborated below.

Through literature screening, 16 studies were finally included, which explored the efficacy of FiLaC in the treatment of different types of anal fistula (based on parks classification).

### Simple vs. complex

**Fig. 3:**
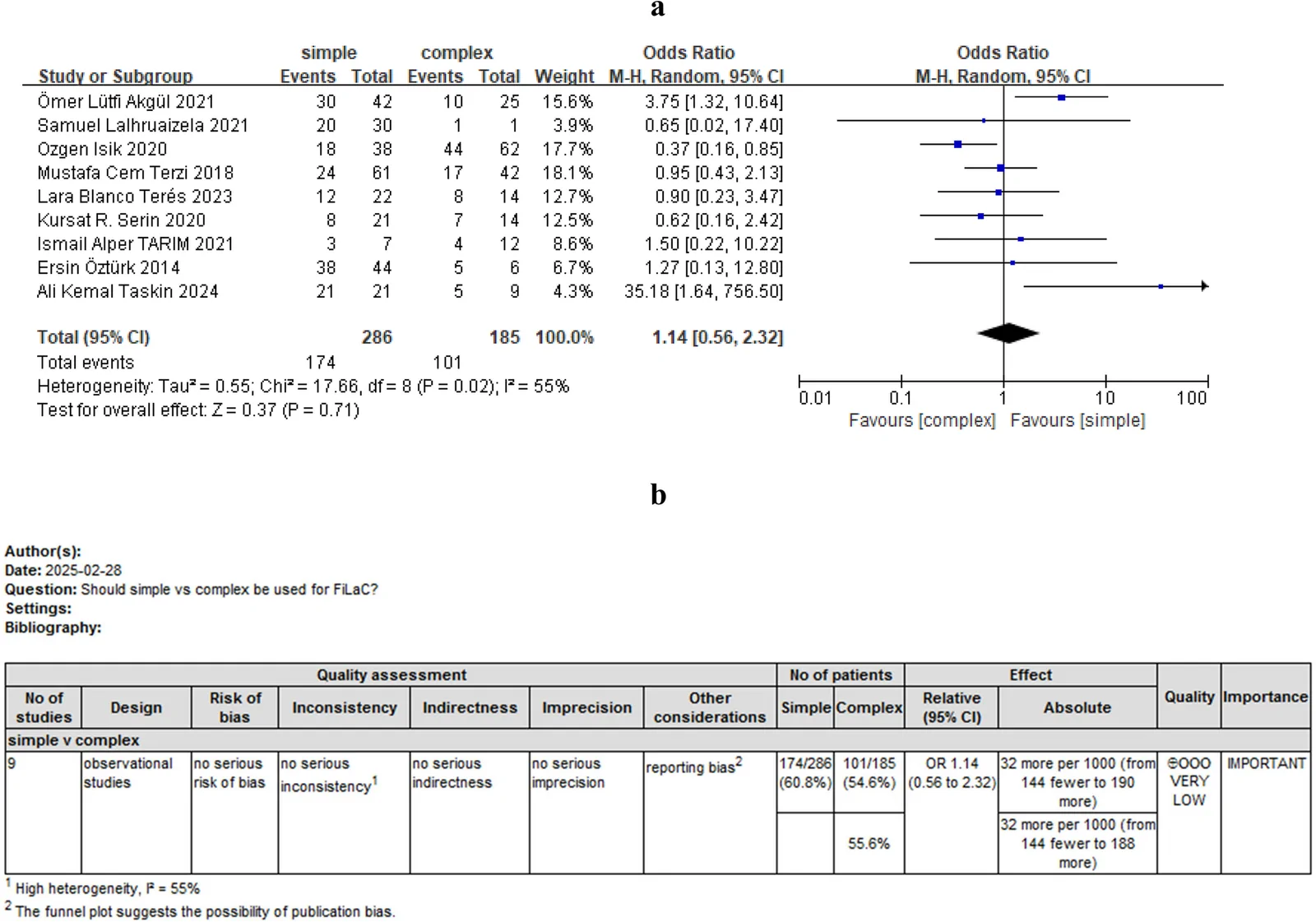
**a**. There was no statistically significant difference between simple and complex fistulae following FiLaC. **b**. Quality assessment for simple vs. complex fistula indicating a very low level of evidence

According to the ASCRS clinical guidelines, complex anal fistulas include trans-sphincteric fistulae that involve greater than 30% of the external sphincter, suprasphincteric, extrasphincteric, or horseshoe fistulas, and anal fistulas associated with inflammatory bowel disease, radiation, malignancy, preexisting fecal incontinence, or chronic diarrhea. Recurrent or branching fistulas are also classified as complex [6]. Most studies did not clearly report the proportions of high and low transsphincteric fistulae or provided detailed classifications for patients with multiple tracts. Pooled results from nine studies indicated healing rates of 60.8% (174/286) vs. 54.6% (101/185) for simple vs. complex fistulae respectively, Fig. 3a and b. This difference was not statistically significant (*p* = 0.71).

However, when comparing the success rates for intersphincteric and transsphincteric fistulae with those of suprasphincteric and extrasphincteric fistulae, a statistically significant difference was observed.

### Intersphincteric / transsphincteric vs. suprasphincteric / extrasphincteric

**Fig. 4:**
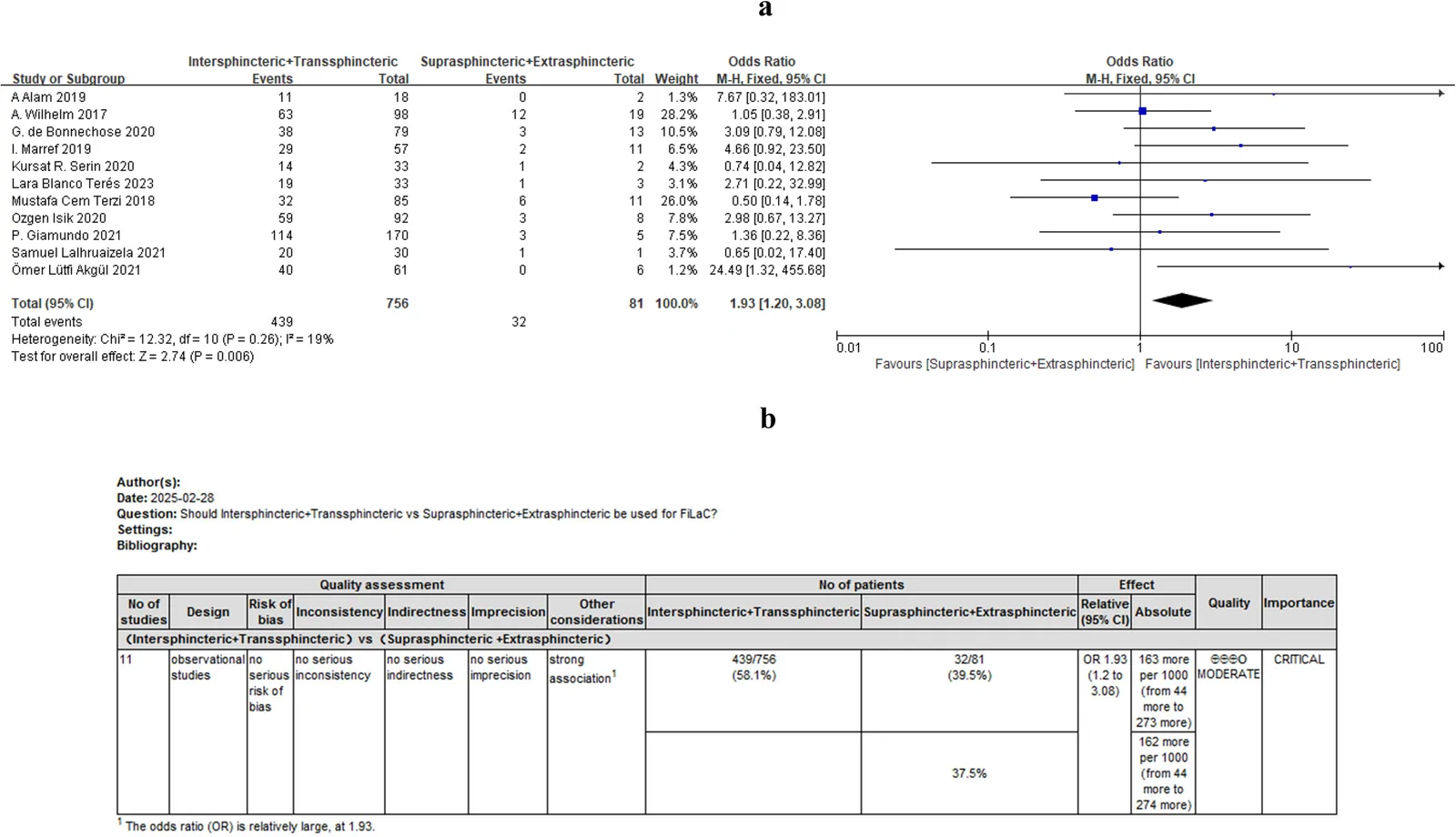
**a**. Intersphincteric & transsphincteric vs. suprasphincteric & extrasphincteric. **b**. Quality assessment for evidence level

Amongst complex fistulae, suprasphincteric and extrasphincteric are even more challenging to manage. Thus, a subgroup analysis was performed to study this specific aspect. Pooled data from 11 studies including 756 patients with intersphincteric or transsphincter fistulae and 81 patients with suprasphincteric and extrasphincteric fistulae, showing a significantly higher odds of healing for the intersphincteric / transsphincteric fistulae in comparison to suprasphincteric / extrasphincteric fistulae, OR: 1,93, *p* = 0.006, Figs. 4a, with a moderate evidence level following quality assessment, Fig. 4b.

### Intersphincteric vs. transsphincteric

**Fig. 5:**
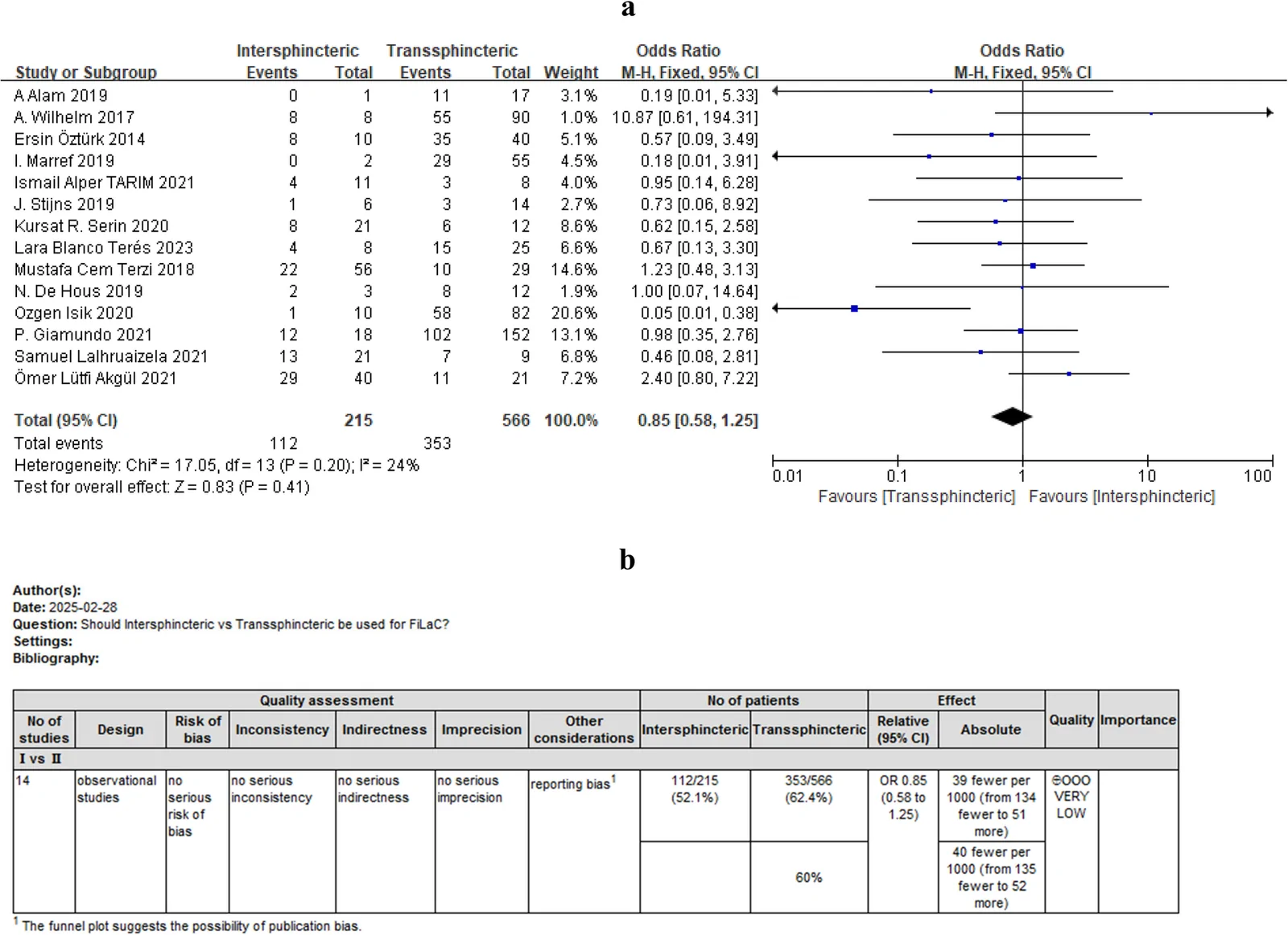
**a**. Intersphincteric vs. transsphincteric fistula. **b**. Quality assessment for evidence level

Pooled data from 14 studies including 607 patients with intersphincteric (164) vs. transsphincteric (443) fistulae indicated success rates of 52.1% vs. 62.4% for intersphincteric vs. transsphincteric fistulae, respectively. The result was not statistically significant (*p* = 0.41), Fig. 5a with a very low quality of evidence, Fig. 5b.

**Fig. 6:**
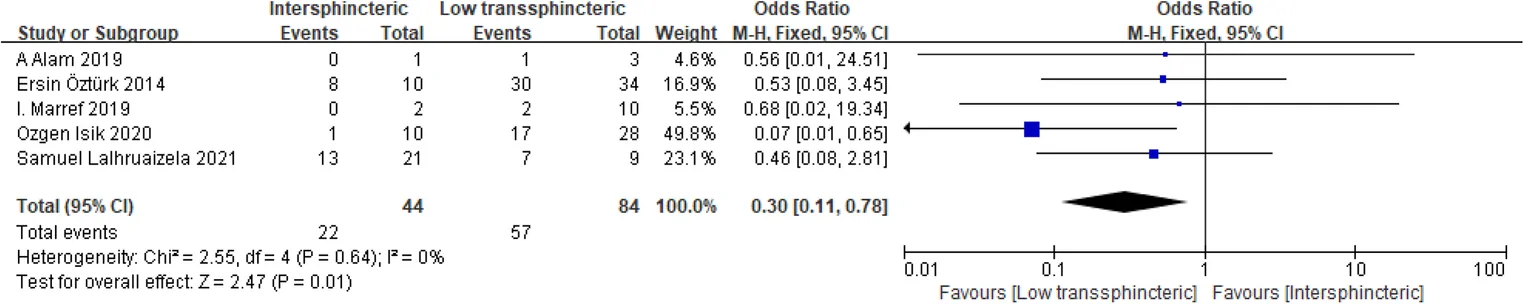
Intersphincteric vs. low transsphincteric fistula. The healing rate following FiLaC was significantly higher for low transsphincteric compared to intersphincteric fistulae

**Fig. 7:**
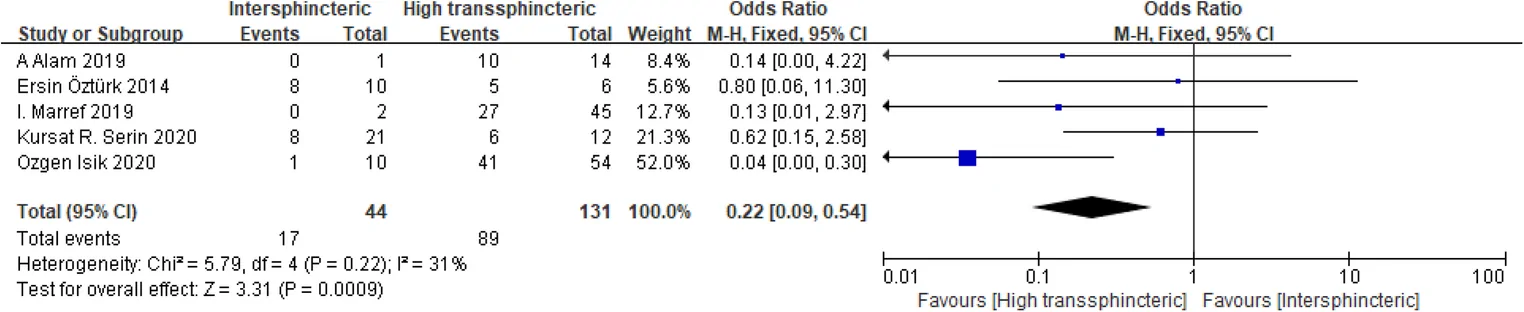
Intersphincteric vs. high transsphincteric Fistula. High transsphincteric fistulae have a significantly higher closure rates following FiLaC compared to intersphincteric fistulae

Further stratification of transsphincteric fistula in low and high did not alter the trend as both low and high transsphincteric fistulae resulted in higher healing rates in comparison to intersphincteric fistulae, Figs. 6 and 7.

### Intersphincteric vs. suprasphincteric

**Fig. 8:**
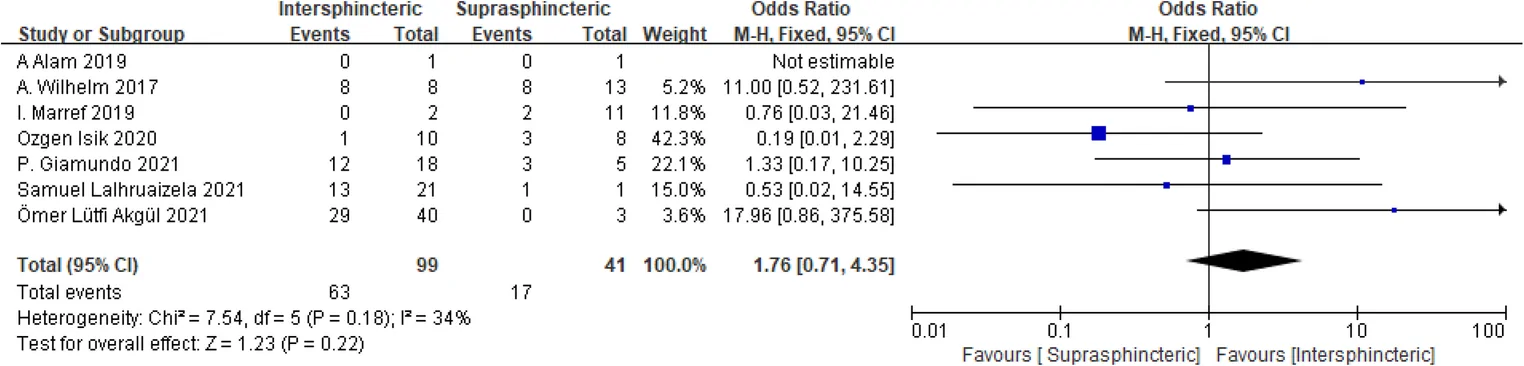
Intersphincteric vs. suprasphincteric fistulae. There was no significant difference in healing rates for intersphincteric vs. suprasphincteric fistulae following FiLaC

Seven studies reported on outcomes of FiLaC for intersphincteric (100) vs. suprasphincteric (42), with healing rates of 63% and 40.5%, respectively. Since none of both fistula types healed in study Alam et al. from 2019, this study was excluded from the meta-analysis. The difference in healing rates between the two types of fistulae was not statistically significant. *p* = 0.22, Fig. 8.

### Intersphincteric vs. extrasphincteric

**Fig. 9:**
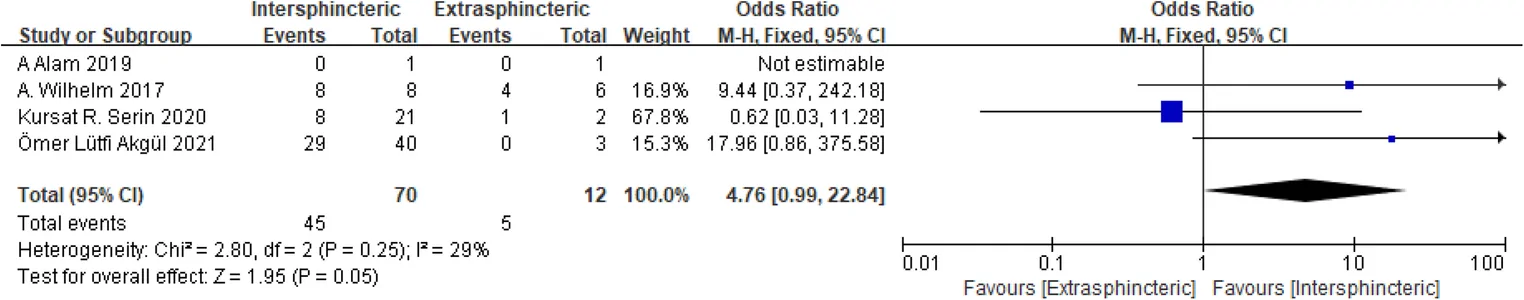
Comparison between intersphincteric and extrasphincteric fistulae showed no statistically significant difference in healing rates

Four studies including 80 patients reported success rates of 64.3% vs. 41.7% for intersphincteric and extrasphincteric fistulae, respectively. Pooled analysis suggests that there is no statistically significant difference in healing between intersphincteric and extrasphincteric fistulae following FiLaC (*p* = 0.05), Fig. 9.

### Low transsphincteric vs. high transsphincteric

**Fig. 10:**
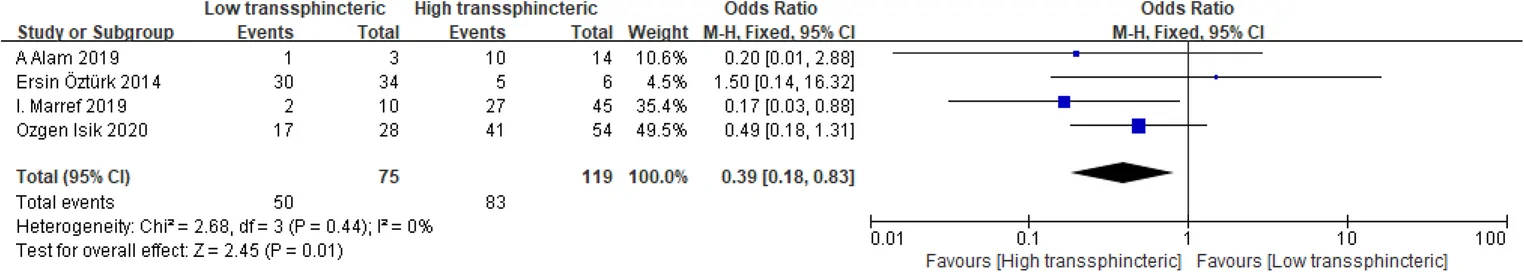
Low vs. high transsphincteric fistula. The healing rate of high transsphincteric fistulae was significantly higher compared to low transsphincteric fistulae following FiLaC

The high of transsphincteric fistulae was reported in four studies including 194 cases, with a closure rate of 66.7% (50/75) vs. 69.7% (83/119) for low vs. high transsphincteric fistula respectively, *p* = 0.01, Fig. 10.

### Transsphincteric vs(suprasphincteric + extrasphincteric)

**Fig. 11:**
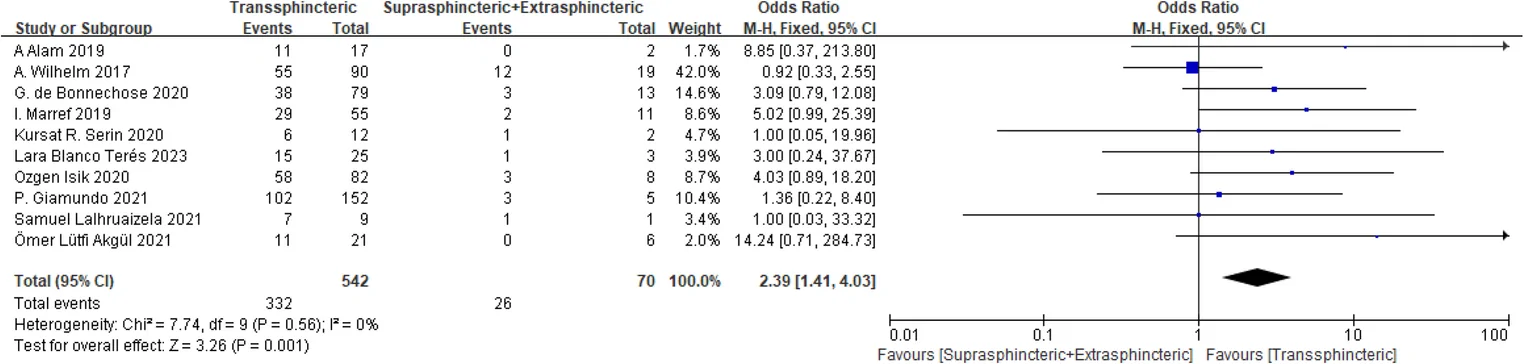
Transsphincteric vs. Suprasphincteric + Extrasphincteric. Transsphincteric fistulae had a significantly higher success rate in comparison to extrasphincteric and suprasphincteric fistulae

Due to the rarity of suprasphincteric and extrasphincteric fistulae, some studies combined them into a single category for analysis. By comparing the cure rates of transsphincteric fistulae with suprasphincteric and extrasphincteric fistulae, the results showed that transsphincteric fistulae had a significantly higher cure rate (OR = 2.39, *P* = 0.001). However, Egger’s test indicated the presence of publication bias (*P* = 0.032). Wilhelm et al. reported cure rates of 61.5% and 66.7% for suprasphincteric and extrasphincteric anal fistulas treated with FiLaC, respectively [41]. A. Alam [27] and Ömer Lütfi Akgül [28] each reported one case of Type III and Type IV anal fistulas, respectively, but neither achieved a cure, Fig. 11.

### Transsphincteric vs. suprasphincteric

**Fig. 12:**
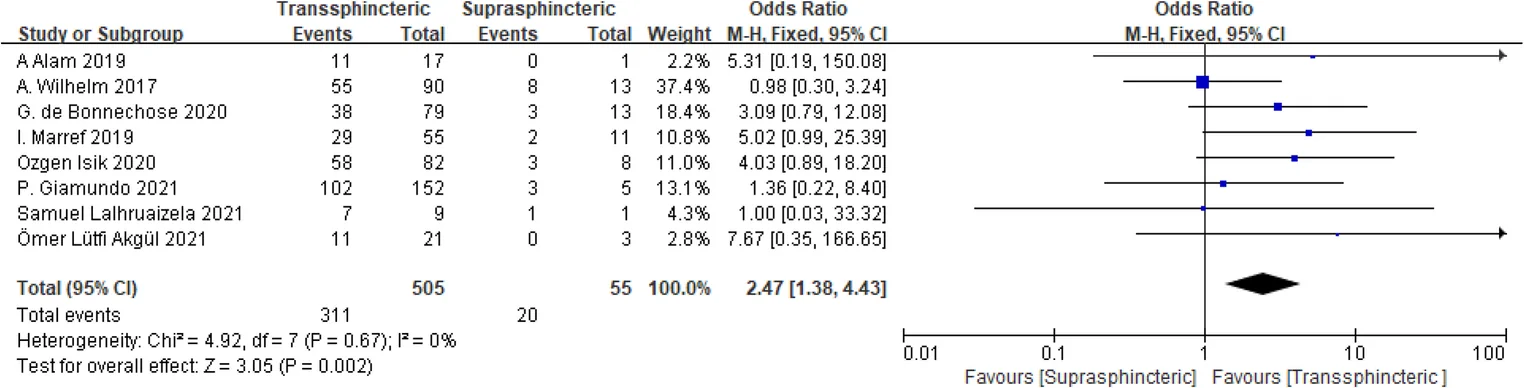
Transsphincteric vs. suprasphincteric fistulae

Eight studies reported on transsphincteric (505) or suprasphincteric (55) fistula course, with closure rates of 61.6% vs. 36.4% for transsphincteric and suprasphincteric fistulae, respectively. The results indicated that the cure rate of transsphincteric fistulas was significantly higher (OR = 2.47, *P* = 0.002), Fig. 12.

### Transsphincteric vs. extrasphincteric

**Fig. 13:**
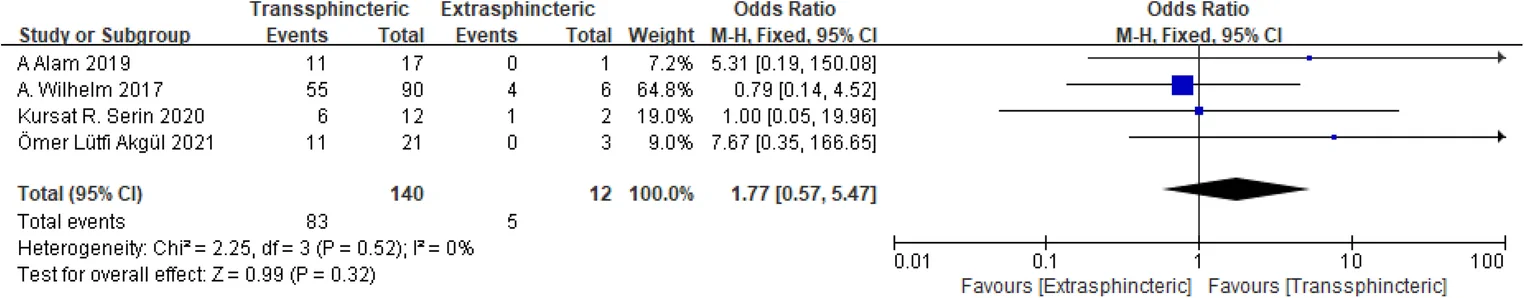
Transsphincteric vs. extrasphincteric fistula

Four studies reported on transsphincteric (140) vs. extrasphincteric (12) course of the fistula tract, indication 59.3% success rate for transsphincteric vs. 41.7% for extrasphincteric fistula, *p* = 0.32, Fig. 13.

## Discussion

This manuscript investigated the efficacy of FiLaC in different types of fistulae based on Park´s classification. In the comparison of efficacy between simple and complex fistulae, pooled analysis showed a higher success rate for simple fistulae compared to complex fistulae after FiLaC. Amongst complex fistulae, the odds of healing following one FiLaC attempt was two times higher in cases with transsphincteric fistulae compared to suprasphincteric / extrasphincteric fistulae. Nevertheless, one half of the suprasphincteric / extrasphincteric fistulae closed following one FiLaC attempt.

Pooled data from meta-analysis indicated a higher success rate for simple fistulae compared to complex fistulae following one FiLaC attempt. A similar finding was reported in a retrospective study from Spain by Blanco Terés et al. [26]. Traditionally, simple fistulae are amenable to fistulotomy with good healing rates and presumed low risk of continence impairment [42, 43]. However, a recent systematic review and meta-analysis by Litta et al. reported a 12.5% pooled rate of continence disturbance following fistulotomy for simple fistulae [44]. Besides, it has been confirmed that so called simple fistulae are not always simple [45]. More so, the complexity of simple fistulae involves more than just the simple fistula anatomy. From a clinical standpoint, anatomically simple fistulae like anterior fistulae in female patients, patients with recurrent fistulae, as well patients with reduced resting tone are at a higher risk of continence disturbance following fistulotomy [7], justifying the classification of such fistulae as “complex”. This clinical aspect therefore justifies the use of FiLaC in selected patients with “simple fistulae”.

A recently published systematic review by Duda et al. failed to show any relationship between fistula type per Park´s classification and closure rates following FiLaC [40]. In contrast to the above systematic review, even though not statistically significant, the odds of healing following FiLaC in this study was two times higher for fistula tracts with an intersphincteric or transsphincteric course compared to extrasphincteric and suprasphincteric fistula courses. Amongst these two types, transsphincteric fistulae have a better odds of healing compared to intersphincteric fistulae. This finding is largely in line with the observation reported by Marref et al. indicating higher success rates for transsphincteric fistulae managed with FiLaC [46]. This important finding may be due to the fact that transsphincteric fistulae have a rather straight course of the fistula tract, that are easier to managed compared to a non-straight course in suprasphincteric and extrasphincteric fistulae. This thought may also account for the good healing rates following the LIFT (Ligation of Intersphincteric Fistula Tract) procedure reported by Rojanasakul [13].

Although the results from this study make a strong argument for the use of FiLaC in fistulae with a rather straight course, about one half of suprasphincteric and extrasphincteric fistulae do heal following FiLaC. This is meaningful finding bearing in mind that most cases of FiLaC failure are associated with a downgrading of the fistula to a simpler fistula with may be amendable to fistulotomy [41, 47]. More so, current literature suggests that FiLaC can safely be repeated in such cases with an increase in the odds of closure [20, 48]. This is a very encouraging finding considering the fact that the minimally invasive nature of FiLaC leaves the surrounding tissue with little or no damage.

The lack of high-quality data represents a relevant limitation to this study. In fact, the analysis is based entirely on findings from studies with retrospective design, with well - known limitations. Also, there is a possibility that the chosen search strategy could have missed some relevant publications. More so, only publication in English language were included. Another limitation to the results reported in this study is the short duration of follow-up reported in some articles. Therefore, the findings reported in this working must be interpreted with caution.

Despite the above limitations, the results of the systematic review and meta-analysis provide good data to guide clinical decision-making with regarding the potential success rate of FiLaC for the different kinds of anal fistula.

## Conclusion

Amongst all fistula types, although not statistically significant simple fistulae have a higher odds of healing following FiLaC in comparison to complex fistulae. Amongst complex fistulae, transsphincteric fistulae have the best success rates following FiLaC. Extrasphincteric and suprasphincteric fistulae have about 50% success rate following FiLaC.

## Supplementary Information

Below is the link to the electronic supplementary material.


Supplementary Material 1 (20.0 KB)


## Data Availability

all data supporting the findings of this study are available within the manuscript.
