# Supplementary material for: Efficacy of FiLaC in different types of anal fistula. A systematic review and meta-analysis
Source: Lasers Med Sci. 2026 Sep 1;41(1):209. doi: 10.1007/s10103-026-05012-1 (PMC13534216; doi:10.1007/s10103-026-05012-1)
Supplement: Supplementary file 1 [file 10103_2026_5012_MOESM1_ESM.docx]

**Supplementary Table 1. Quality assessment of included retrospective studies.**

|  | Selection of Study Population | | | | Between-Group Comparability | Outcome Measurement | | | Total Score |
| --- | --- | --- | --- | --- | --- | --- | --- | --- | --- |
| Study |  | ② | ③ | ④ | ⑤ | ⑥ | ⑦ | ⑧ |  |
| P. Giamundo 2021 | 1 | 1 | 1 | 1 | 1 | 1 | 1 | 1 | 8 |
| Lara Blanco Terés 2023 | 1 | 1 | 1 | 1 | 1 | 1 | 1 | 1 | 8 |
| A. Alam 2019 | 1 | 1 | 1 | 1 | 1 | 1 | 0 | 1 | 7 |
| Ömer Lütfi Akgül 2021 | 1 | 1 | 1 | 1 | 1 | 1 | 1 | 1 | 8 |
| Samuel Lalhruaizela 2021 | 1 | 1 | 1 | 1 | 1 | 1 | 1 | 1 | 8 |
| Kursat R. Serin 2020 | 1 | 1 | 1 | 1 | 1 | 1 | 1 | 1 | 8 |
| G. de Bonnechose 2020 | 1 | 1 | 1 | 1 | 1 | 1 | 0 | 1 | 7 |
| I. Marref 2019 | 1 | 1 | 1 | 1 | 2 | 1 | 0 | 1 | 8 |
| A. Wilhelm 2017 | 1 | 1 | 1 | 1 | 1 | 1 | 1 | 1 | 8 |
| Ozgen Isik 2020 | 1 | 1 | 1 | 1 | 1 | 1 | 1 | 1 | 8 |
| J. Stijns 2019 | 1 | 1 | 1 | 1 | 1 | 1 | 1 | 1 | 8 |
| N. De Hous 2019 | 1 | 1 | 1 | 1 | 1 | 0 | 0 | 1 | 6 |
| Mustafa Cem Terzi 2018 | 1 | 1 | 1 | 1 | 1 | 1 | 1 | 1 | 8 |
| Ersin Öztürk 2014 | 1 | 1 | 1 | 1 | 1 | 1 | 1 | 1 | 8 |
| Ismail Alper TARIM 2021 | 1 | 1 | 1 | 1 | 1 | 1 | 0 | 1 | 7 |

Fifteen cohort studies were assessed for quality using the NOS（Newcastle-Ottawa Scale） scale, which includes 8 items with a maximum score of 9 points.

Selection of Study Population：① Representativeness of the Exposed Group；② Selection Method for the Non-Exposed Group；③ Method for Determining Exposure Factors；④ Confirmation of No Outcome of Interest at Study Initiation；

Between-Group Comparability：⑤ Consideration of Comparability Between Exposed and Non-Exposed Groups in Design and Statistical Analysis；

Outcome Measurement：⑥ Adequacy of Outcome Evaluation in the Study；⑦ Sufficient Duration of Follow-Up After Outcome Occurrence；⑧ Adequacy of Follow-Up for Exposed and Non-Exposed Groups

**Supplementary Table 2**. **Quality assessment of the included cross-sectional study.**

| Study |  |  |  |  |  |  |  |  |  |  | ⑪ | Total Score |
| --- | --- | --- | --- | --- | --- | --- | --- | --- | --- | --- | --- | --- |
| Ali Kemal Taskin 2024 | Yes | Yes | Yes | Yes | Yes | Yes | Yes | Yes | No | No | No | 8 |

A descriptive cross-sectional study was assessed for quality using the AHRQ (Agency for Healthcare Research and Quality scale) scale, which consists of 11 items. ① Define the source of information (survey, record review)；② List inclusion and exclusion criteria for exposed and unexposed subjects (cases and controls) or refer to previous publications；③ Indicate time period used for identifying patients；④ Indicate whether or not subjects were consecutive if not population-based；⑤ Indicate if evaluators of subjective components of study were masked to other aspects of the participants；⑥ Describe any assessments undertaken for quality assurance purposes (e.g., test/retest of primary outcome measurements)；⑦ Explain any patient exclusions from analysis；⑧ Describe how confounding was assessed and/or controlled；⑨ If applicable, explain how missing data were handled in the analysis；⑩ Summarize patient response rates and completeness of data collection；⑪ Clarify what follow-up, if any, was expected and the percentage of patients for which incomplete data or follow-up was obtained.
